# Mapping Research on Virtual Reality for Balance, Coordination, and Motor Rehabilitation: A Bibliometric Analysis with Topic Modeling

**DOI:** 10.3390/healthcare14081067

**Published:** 2026-04-17

**Authors:** Hongfei Zhang, Wenjun Hu, Qing Zhang, Man Jiang, Jakub Kortas

**Affiliations:** Department of Health and Life Sciences, Gdansk University of Physical Education and Sport, 80-336 Gdańsk, Poland; zhanghongfei77@126.com (H.Z.); wenjunhusport@163.com (W.H.); qing.zhang@awf.gda.pl (Q.Z.); man.jiang@awf.gda.pl (M.J.)

**Keywords:** virtual reality, balance, coordination, motor rehabilitation, topic modeling

## Abstract

Virtual reality (VR) has been increasingly adopted as a digital tool in rehabilitation for balance training, coordination improvement, and motor recovery, yet the literature remains dispersed across clinical rehabilitation, exercise-based interventions, and broader motor-related applications. This fragmentation makes it difficult to determine how the field has evolved and where research emphasis has shifted. This study mapped the research landscape and thematic evolution of VR for balance, coordination, and motor rehabilitation using bibliometric analysis and topic modeling. A total of 1258 articles indexed in the Web of Science Core Collection from 2011 to 2025 were analyzed. Only English language articles and reviews relevant to VR-based balance, coordination, or motor rehabilitation research were included, yielding a final dataset of 1258 publications. CiteSpace and VOSviewer were used to examine keyword co-occurrence, clustering patterns, and temporal trends, while Latent Dirichlet Allocation (LDA) was applied to identify latent themes and their temporal dynamics. The field has moved beyond early feasibility testing toward a more differentiated landscape shaped by distinct clinical targets, population groups, and training purposes. Seven recurring themes were identified, including vestibular rehabilitation and immersive training, post-stroke upper-limb rehabilitation, efficacy and adverse-effect assessment, balance and gait training interventions, evidence synthesis and review-based evaluation, elderly exercise and cognitive interventions, and skill-oriented virtual task training with recent expansion toward broader population groups and task-specific applications beyond traditional rehabilitation settings. VR research on balance, coordination, and motor rehabilitation has evolved into a more thematically differentiated field rather than remaining a single rehabilitation-oriented domain. By combining bibliometric mapping with topic modeling, this study clarifies where evidence is concentrated and which thematic directions are gaining visibility, providing a clearer basis for future evidence synthesis and more comparable intervention reporting.

## 1. Introduction

Virtual reality (VR) has been increasingly used in balance training, coordination enhancement, and motor rehabilitation because it enables interactive, task-oriented practice in controllable environments and allows feedback to be delivered immediately and adaptively. These features are particularly relevant in motor rehabilitation, where structured repetition, graded task difficulty, and timely feedback are central to motor relearning and functional recovery [1,2,3,4]. Compared with conventional exercise- and equipment-based approaches, VR can provide more individualized and context-rich training while also supporting engagement through immersive and game-like interaction [5,6,7]. As a result, VR has attracted growing interest across a broad range of movement-related and rehabilitation applications [8,9,10].

Recent studies have applied VR across a wide range of motor-related contexts, including postural and mobility training, upper-limb rehabilitation, sensory–motor rehabilitation, and cognitively enriched movement interventions. Some work also extends to skill-oriented training in younger and other non-clinical populations. Controlled trials have reported improvements in outcomes such as postural control, balance performance, and task-specific motor function, while neurophysiological and neuroimaging research has begun to explore the mechanisms that may underlie VR-based practice [11,12,13,14,15]. At the same time, the literature has expanded across clinical rehabilitation, exercise-based intervention, motor learning, and broader sport-related contexts. This growth has increased both the visibility and diversity of the field, but it has also made the evidence base more dispersed. As a result, it has become more difficult to determine how research priorities have shifted over time, where evidence is most concentrated, and which lines of work are emerging.

This challenge is not fully addressed by existing reviews alone. Recent bibliometric studies have also explored VR-related rehabilitation themes in specific populations, including older adults [16]. Although previous narrative and descriptive reviews have provided valuable summaries of VR interventions in specific populations, clinical conditions, or functional domains, they are typically structured around narrower populations, intervention aims, or outcome categories. As a result, they are less able to capture how the broader field has evolved across multiple populations, intervention purposes, and application settings, or to clarify where evidence is most concentrated and which research lines are gaining visibility over time. In a literature base that now spans rehabilitation, exercise-based intervention, motor learning, and broader training-oriented contexts, a more field-level perspective is needed [17,18,19].

Bibliometric approaches can help address this limitation by mapping publication output, keyword relationships, network structures, and temporal patterns at the macro level. However, bibliometric mapping alone is less suited to identifying the latent semantic structure of a corpus or to explaining how recurring research themes are organized and shift over time. Topic-modeling methods, in contrast, can detect underlying thematic patterns across large text datasets, but they do not by themselves reveal the relational and developmental structure that bibliometric analysis makes visible. When used together, these approaches provide complementary insight by linking the observable structure of a research field with its underlying thematic organization. This combined strategy is therefore particularly suitable for a field such as VR-based balance, coordination, and motor rehabilitation, where the literature has expanded rapidly but remains conceptually dispersed across multiple clinical and non-clinical domains.

Against this background, the present study integrates bibliometric mapping (CiteSpace and VOSviewer) with Latent Dirichlet Allocation (LDA) topic modeling to examine VR research related to balance, coordination, and motor rehabilitation from 2011 to 2025. Specifically, this study aims to (1) characterize publication trends and developmental phases, (2) identify established, growing, and emerging research themes, and (3) clarify how the field has become differentiated across population groups, intervention purposes, and training contexts. By combining macro-level mapping with topic-level semantic analysis, this study seeks to provide a more structured overview than conventional descriptive summaries and to offer a clearer basis for future evidence synthesis, more comparable intervention reporting, and more coherent development of VR applications in rehabilitation and motor training.

## 2. Materials and Methods

### 2.1. Search Strategy and Data Retrieval

**Figure 1:**
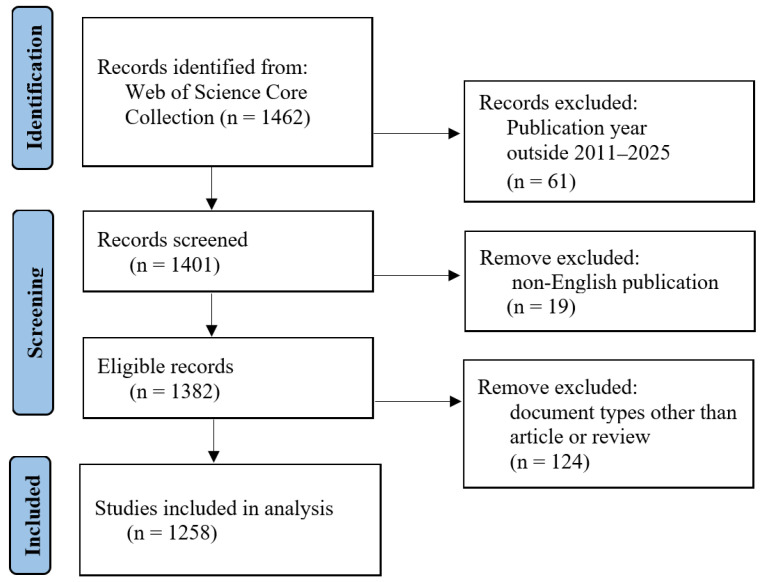
Flowchart of literature screening and study selection.

The literature search was conducted in the Web of Science Core Collection (WoSCC) using an advanced search interface. WoSCC was selected as the sole data source because it provides standardized bibliographic records, consistent indexing, and complete citation information, which are well suited to integrated bibliometric mapping and topic-modeling analysis. It is also a commonly used source in bibliometric research because its standardized indexing and citation-linking structure support reliable citation-based analyses and network visualization. Boolean/phrase expanders were applied to ensure comprehensive retrieval of relevant studies, and filters were set to exclude records that were not peer-reviewed or not published in English. The search string in the “Topic” field combined three concept clusters with the operators “OR” and “AND” as follows: (“virtual reality” OR VR OR exergaming OR “VR-based intervention” OR “VR exercise”) AND (“balance” OR “coordination” OR “balance impairment” OR “balance deficit” OR “coordination impairment” OR “coordination deficit” OR “motor function” OR “motor dysfunction” OR motor rehabilitation) AND (“sport” OR “exercise”). The “sport” OR “exercise” cluster was included to focus the dataset on exercise-, training-, and movement-oriented uses of VR in relation to balance, coordination, and motor function. The initial query retrieved 1462 records. Selection criteria were applied as follows: (1) Document type limited to “Article” or “Review”; (2) Publication date between January 2011 and December 2025; (3) Documents are published in English. After applying these criteria, 1258 records remained. These records were then reviewed for topical relevance at the title/abstract level, and no additional records were excluded at this stage. The screening and eligibility process is summarized in Figure 1.

### 2.2. Eligibility Criteria

The retrieved records were screened in two stages. First, titles and abstracts were checked to remove records that were clearly outside the scope of VR-related balance, coordination, or motor-function-oriented exercise and rehabilitation research. Second, the remaining records were reviewed against the predefined eligibility criteria, including document type, language, publication period, and topical relevance to the study scope. The screening process was conducted independently by two evaluators, and any disagreements were resolved through discussion; when necessary, a third evaluator reviewed the record and made the final decision. The final corpus consisted of 1258 publications and was used as the unified dataset for all subsequent analyses.

### 2.3. Data Analyses

The 1258 included publications were analyzed through an integrated multi-step workflow. First, the dataset was imported into CiteSpace (Version 6.1.R6, Drexel University, Philadelphia, PA, USA) to generate keyword co-occurrence networks, calculate betweenness centrality, perform log-likelihood ratio (LLR) clustering, detect keyword bursts, and visualize temporal patterns through timeline and time-zone mapping. Second, VOSviewer (Version 1.6.17, Centre for Science and Technology Studies, Leiden University, Leiden, The Netherlands) was used to conduct secondary visualization of the co-occurrence structure, optimize node layout, and assist in cluster interpretation and topic naming. Third, descriptive statistics and publication-trend visualizations were produced in R (Version 4.2.0, R Foundation for Statistical Computing, Vienna, Austria) using the bibliometric, igraph, and ggplot2 packages. Finally, Latent Dirichlet Allocation (LDA) topic modeling was performed on the same corpus to identify latent semantic themes. Before topic modeling, the textual corpus was preprocessed through tokenization, lowercasing, removal of punctuation and stopwords, part-of-speech tagging, and lemmatization. Only informative terms composed of alphabetic characters and longer than three letters were retained for topic extraction. Topic-number evaluation was conducted using coherence and perplexity, and the final topic solution was selected by prioritizing semantic interpretability when model fit remained comparable across nearby values of K. Candidate topic numbers from K = 2 to K = 15 were evaluated using perplexity and coherence (C_v), and K = 7 was selected as the optimal solution by balancing semantic interpretability and model robustness. Based on this model, topic keywords, topic prevalence, temporal evolution, and topic–keyword relationships were further analyzed and visualized. This integrated workflow enabled the study to examine both the macro-level structural features of the literature and the micro-level evolution of its thematic content within a single analytical framework.

## 3. Results

### 3.1. Keyword-Based Thematic Structure and Temporal Evolution

**Figure 2:**
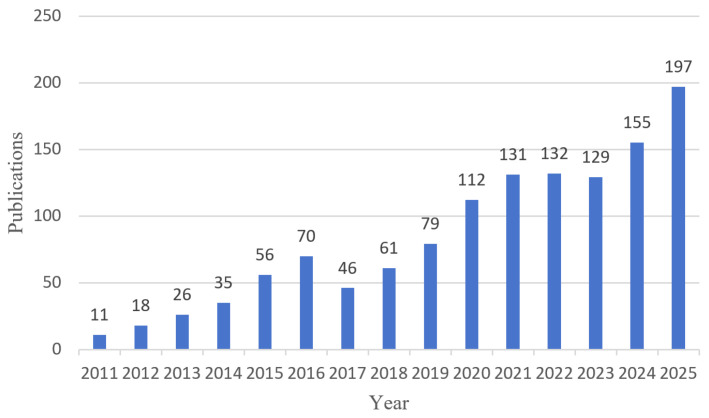
Annual publication trend of studies published between 2011 and 2025.

This section examines the thematic structure and temporal evolution of the field based on keyword-level bibliometric evidence, with particular attention to publication trends, thematic differentiation, and changing research emphasis across the literature. Using CiteSpace, we analyzed keyword co-occurrence, cluster structure, burst detection, and time-zone mapping to identify major research foci and their shifts over time. Figure 2 shows the annual publication trend in VR research on balance, coordination, and motor rehabilitation from 2011 to 2025. Overall, the number of publications increased over time, indicating sustained growth in this research field.

#### 3.1.1. Keyword Analysis

**Figure 3:**
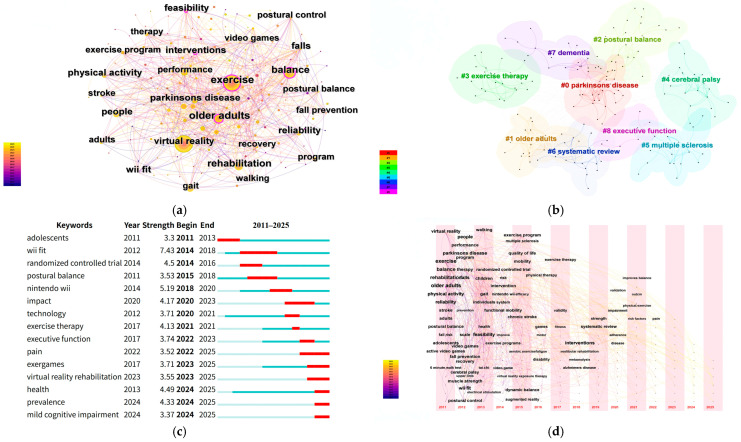
Keyword Analysis. (**a**) Construction of Keyword Co-occurrence Network; (**b**) Topic Clustering Features and Cluster Label Extraction; (**c**) Keywords with the Strongest Citation Bursts; (**d**) The evolution trajectory of themes under the time zone map.

A total of 196 keywords were extracted to construct the co-occurrence network (Figure 3a). The most frequently occurring keywords were “virtual reality” (*n* = 549), “exercise” (*n* = 377), “balance” (*n* = 327), “rehabilitation” (*n* = 226), and “older adults” (*n* = 193), indicating that the field is centered on VR-supported exercise and rehabilitation, with particular emphasis on balance-related outcomes and aging populations. In terms of betweenness centrality, the most influential bridging keywords were “older adults” (0.13), “balance” (0.12), “feasibility” (0.12), “exercise” (0.11), and “physical activity” (0.10). These terms appear to connect otherwise distinct research strands, suggesting that population characteristics, intervention feasibility, and exercise-based applications play an important integrative role in the knowledge structure of the field (Table 1).

The keyword cluster map further showed that the literature is organized around several recurrent application domains, including balance-related rehabilitation, neurological conditions, older-adult interventions, and evidence synthesis (Figure 3b). Taken together, the co-occurrence and clustering results suggest that the field has developed around a core rehabilitation-and-exercise axis, while gradually expanding into more differentiated clinical and population-specific contexts.

From a temporal perspective, the evolution of keywords indicates a clear shift in research emphasis over time (Figure 3c,d). Between 2011 and 2015, the literature was primarily shaped by broad foundational terms such as “virtual reality”, “exercise”, “balance”, and “physical activity”, reflecting an early stage focused on feasibility, general intervention potential, and basic application framing. From 2016 to 2020, more specific terms such as “intervention”, “systematic review”, and “vestibular rehabilitation” became increasingly visible, suggesting a transition toward structured evaluation, evidence synthesis, and more targeted rehabilitation contexts. Since 2021, newer terms such as “improves balance”, “validation”, “pain”, “exergames”, and “virtual reality rehabilitation” have emerged, indicating a further movement toward outcome-oriented assessment, applied validation, and more specialized intervention scenarios. Overall, the keyword evidence suggests that research in this field has progressed from early feasibility-oriented exploration to a more differentiated and application-specific stage [20,21,22].

#### 3.1.2. Country and Institution Analysis

**Figure 4:**
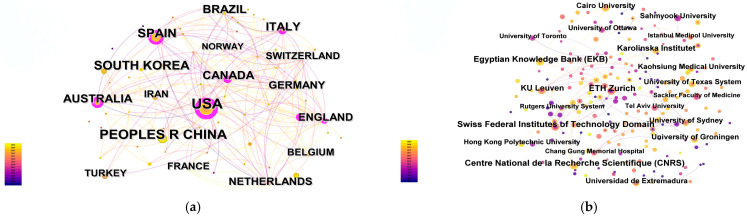
Collaboration networks of countries (**a**) and institutions (**b**) in VR research on balance, coordination, and motor rehabilitation. Node size reflects publication output, and links indicate collaboration relationships.

The country collaboration network shown in Figure 4a (67 nodes, 205 edges; network density = 0.0927) illustrates the international distribution of research activity in this field. The United States occupies the most central position in the network and appears to be the leading hub of international collaboration. China, Spain, and Australia also show strong research visibility and form an active group of highly connected contributors. In addition, Canada and England exhibit relatively high betweenness centrality, suggesting that they play an important bridging role in linking research groups across North America and Europe. Overall, the country-level network indicates that the field has developed through a relatively concentrated but internationally connected pattern of collaboration.

The institutional collaboration network shown in Figure 4b (205 nodes, 127 edges; network density = 0.0061) is notably more dispersed, indicating a lower overall level of inter-institutional connectivity. Among the institutions, ETH Zurich (Swiss Federal Institutes of Technology Domain) appears as the most prominent node in terms of both publication output and network centrality, suggesting a leading role in cross-institutional collaboration. Centre National de la Recherche Scientifique (CNRS) and KU Leuven also occupy important positions in the network and appear to contribute substantially to multinational research linkages. Other institutions, including the University of Toronto, Hong Kong Polytechnic University, and the University of Sydney, also show visible collaborative roles, particularly as connecting nodes within broader international partnerships. Taken together, these findings suggest that while country-level collaboration is relatively well connected, institutional collaboration remains more fragmented and concentrated around a limited number of influential hubs.

#### 3.1.3. Document and Journal Analysis

**Figure 5:**
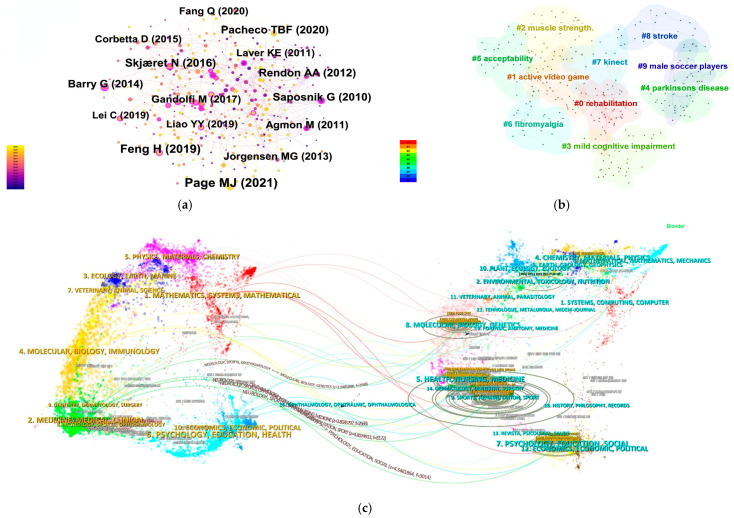
Document and Journal Analysis. (**a**) Document co-citation network; (**b**) co-citation clustering structure; (**c**) Dual-map overlay of journals.

The document co-citation network shown in Figure 5a (367 nodes, 510 edges; density = 0.0076) highlights the foundational literature underpinning this field. Highly cited and structurally central references occupy prominent positions in the network, suggesting that the field is built on a core set of studies that shaped early evidence on VR-based exercise, rehabilitation, and balance-related interventions. Their central location within the co-citation structure further indicates that they serve as key reference points linking subsequent studies across multiple application contexts.

The co-citation clustering results shown in Figure 5b demonstrate a well-structured knowledge base (Modularity Q = 0.754; mean silhouette = 0.9257). A total of 10 clusters were identified, among which #0 rehabilitation (*n* = 49), #1 active video game (*n* = 46), #2 muscle strength (*n* = 44), #3 mild cognitive impairment (*n* = 42), and #4 Parkinson’s disease (*n* = 40) were the largest. These major clusters indicate that the co-cited literature is organized around several recurring lines of work, including rehabilitation-focused applications, game-based intervention approaches, physical function training, cognitive-related assessment and intervention, and neurological conditions. Overall, the clustering pattern suggests that the field is supported by a diverse but coherent evidence base that spans both clinical rehabilitation and broader function-oriented intervention contexts.

The dual-map overlay of journals shown in Figure 5c further illustrates the interdisciplinary dissemination pattern of the field. The citing literature is concentrated primarily in “Medicine, Medical, Clinical” and “Psychology, Education, Health”, whereas the cited literature is distributed across broader disciplinary domains, particularly “Health, Nursing, Medicine” and “Molecular, Biology, Genetics”. The main citation paths indicate that research in this area draws simultaneously on clinical, behavioral, and biomedical knowledge streams. This pattern suggests that VR research for balance, coordination, and motor rehabilitation has developed through sustained interaction between applied clinical research, health-related behavioral science, and underlying biomedical evidence.

### 3.2. LDA-Based Topic Modeling and Evolutionary Trend Analysis

#### 3.2.1. Perplexity and Coherence Evaluation

**Figure 6:**
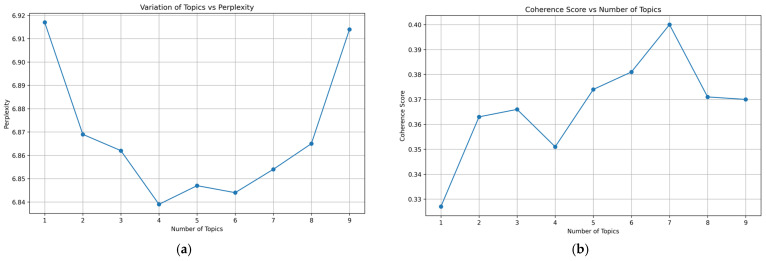
Perplexity and Coherence Evaluation. (**a**) Perplexity vs. Number of Topics; (**b**) Coherence C_v vs. Number of Topics.

To determine the optimal number of topics for the LDA model, candidate models with different topic numbers were evaluated using both perplexity and coherence (C_v) (Figure 6). As the number of topics increased, the coherence score rose overall and reached its highest value at K = 7 (approximately 0.40), indicating the strongest semantic interpretability among the tested solutions. Although perplexity reached its minimum at K = 4 (approximately 6.84), the difference between K = 4 and K = 7 (approximately 6.85) was small and did not indicate a meaningful advantage for the lower-topic solution. In the present study, topic-number selection did not rely on perplexity alone. When perplexity remained comparably stable across nearby values of K, greater weight was given to coherence and thematic interpretability. Compared with K = 4, the K = 7 solution also provided clearer separation among major thematic directions in the corpus. Coherence values in LDA analyses are corpus-dependent and are more appropriately interpreted in relative rather than absolute terms. In applied topic-modeling studies, C_v values in this approximate range are not uncommon for interpretable solutions, depending on corpus characteristics and preprocessing choices. In the present corpus, emphasis was therefore placed on the highest coherence within the tested range together with the semantic clarity of the resulting topic structure. Accordingly, K = 7 was retained for the subsequent topic identification and temporal evolution analyses.

#### 3.2.2. Topic Overview and Analysis

**Figure 7:**
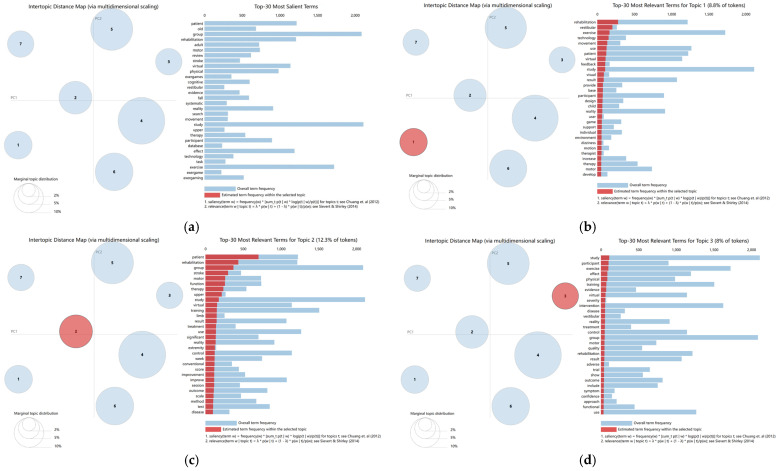
Topic overview and analysis. (**a**) Analysis of the seven topics; (**b**) Vestibular rehabilitation and immersive training; (**c**) Post-stroke upper-limb motor rehabilitation; (**d**) Efficacy and adverse effect assessment. Bubble size represents relative topic prevalence, and distance between bubbles reflects semantic separation among topics. The numbers inside the bubbles indicate topic identifiers. Red marks the selected topic and blue marks the remaining topics; in the bar chart, red indicates estimated term frequency within the selected topic and blue indicates overall term frequency.

**Figure 8:**
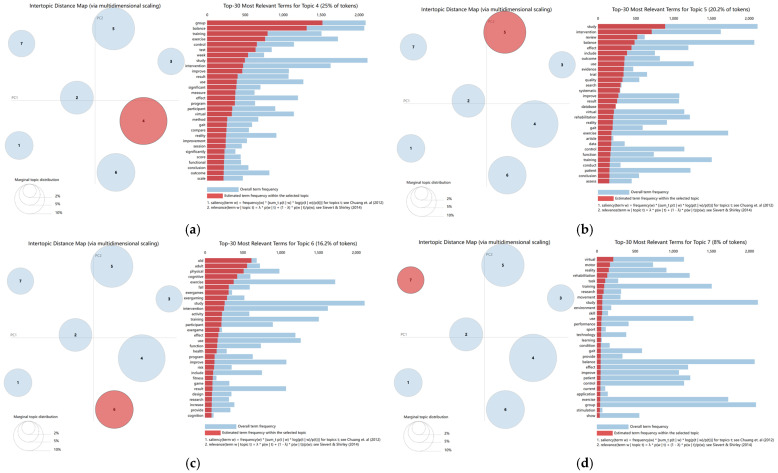
Topic overview and analysis. (**a**) Balance and gait training interventions; (**b**) Evidence synthesis and review-based evaluation; (**c**) Elderly exercise and cognitive interventions; (**d**) Skill-oriented virtual task training. Bubble size represents relative topic prevalence and distance between bubbles reflects semantic separation among topics. The numbers inside the bubbles indicate topic identifiers. Red marks the selected topic and blue marks the remaining topics; in the bar chart, red indicates estimated term frequency within the selected topic and blue indicates overall term frequency.

Based on the optimal topic number (K = 7), the LDA model identified seven recurring themes within the corpus: (1) vestibular rehabilitation and immersive training, (2) post-stroke upper-limb motor rehabilitation, (3) efficacy and adverse-effect assessment, (4) balance and gait training interventions, (5) evidence synthesis and review-based evaluation, (6) elderly exercise and cognitive interventions, and (7) skill-oriented virtual task training (Figure 7 and Figure 8). These topic-level findings complement the bibliometric results by showing how the broader keyword structure is differentiated into more specific lines of rehabilitation-oriented, evaluative, population-specific, and training-related research. Overall, the seven-topic solution suggests a field that has moved beyond a single rehabilitation-centered focus toward a more diversified thematic structure.

Figure 7a provides an overview of the seven-topic structure. In the intertopic distance map, the relative positions of the bubbles reflect semantic differences among topics, whereas bubble size represents the marginal topic distribution within the corpus. Among the seven topics, balance and gait training interventions occupy the largest proportion, indicating that it is the most prominent theme in the dataset. By contrast, vestibular rehabilitation and immersive training and skill-oriented virtual task training appear relatively more separated from the central cluster, suggesting that they represent more specialized or distinct application domains. The corresponding bar charts of salient terms further support the interpretability of the topic structure by showing the most relevant terms associated with each topic [23,24,25].

Topic 1: Vestibular rehabilitation and immersive training (Figure 7b) centers on vestibular-related VR applications, as reflected by representative terms such as “vestibular”, “dizziness”, and “feedback”. This theme highlights individualized and feedback-supported interventions aimed at sensory rehabilitation and balance-related functional improvement [26,27,28,29].

Topic 2: Post-stroke upper-limb motor rehabilitation (Figure 7c) focuses on VR-assisted recovery of upper-limb function after stroke, with representative terms including “stroke”, “upper extremity”, and “therapy”. It reflects a well-established rehabilitation line in which task-specific practice and comparison with conventional treatment remain central [30,31,32].

Topic 3: Efficacy and adverse-effect assessment (Figure 7d) relates to trial-based evaluation of intervention outcomes, as suggested by terms such as “trial”, “adverse”, and “symptom”. This theme indicates that the field has also paid attention to tolerability, symptom monitoring, and the balanced assessment of both therapeutic benefits and potential side effects.

Topic 4: Balance and gait training interventions (Figure 8a) is the largest and most dominant topic in the corpus, represented by terms such as “balance”, “gait”, and “training.” It reflects the central role of postural control and walking-related rehabilitation in the field and shows that structured intervention evaluation has remained a major component of this research area [33,34].

Topic 5: Evidence synthesis and review-based evaluation (Figure 8b) is represented by terms including “review”, “systematic”, and “evidence”. Unlike the more application-specific topics, this theme reflects the growing role of synthesis and quality evaluation, suggesting that the field has accumulated a sufficient body of primary studies to support broader comparative and review-based work.

Topic 6: Elderly exercise and cognitive interventions (Figure 8c) centers on aging-related applications, with representative terms such as “fall”, “exergames”, and “cognitive”. This theme suggests that VR research has expanded beyond rehabilitation alone toward preventive, exercise-based, and multidomain interventions in older adults [35,36,37].

Topic 7: Skill-oriented virtual task training (Figure 8d) reflects more training-oriented VR applications, as indicated by terms such as “skill”, “learning”, and “task”. Compared with the more clinically focused topics, this theme points to a more outward-expanding line of research related to motor learning, performance development, and structured virtual task practice in younger or other non-clinical populations [38,39].

Overall, the seven-topic solution suggests a field that has moved beyond a single rehabilitation-centered focus toward a more differentiated structure across population groups, intervention purposes, and application contexts.

Taken together, the bibliometric and LDA findings suggest a consistent but differently scaled picture of the field. At the bibliometric level, keyword and co-citation patterns highlight broad emphases such as rehabilitation, balance, gait, exercise, and older adults. The LDA results reflect these same broad directions, but further differentiate them into more specific thematic domains—for example, aging-related patterns are reflected more specifically in elderly exercise and cognitive interventions, while broader rehabilitation-oriented patterns are separated into balance and gait training interventions, post-stroke upper-limb rehabilitation, and vestibular rehabilitation and immersive training. In this sense, the bibliometric analyses capture the macro-level structure of the field, whereas the LDA themes clarify how these broader clusters are organized into more focused lines of research.

This integrated view also helps clarify the field’s practical and developmental profile. Population-related patterns are reflected in the prominence of older adults, post-stroke populations, and non-clinical younger groups; intervention-related patterns are reflected in the differentiation between rehabilitation-focused, evaluative, and skill-oriented applications; and future trends are suggested by the growing visibility of aging-related interventions, evidence synthesis, and task- and performance-oriented VR uses beyond traditional rehabilitation settings.

For transparency, the top terms with weights for each LDA topic are provided in Supplementary Table S1.

#### 3.2.3. Topic Prevalence and Strength Analysis

**Figure 9:**
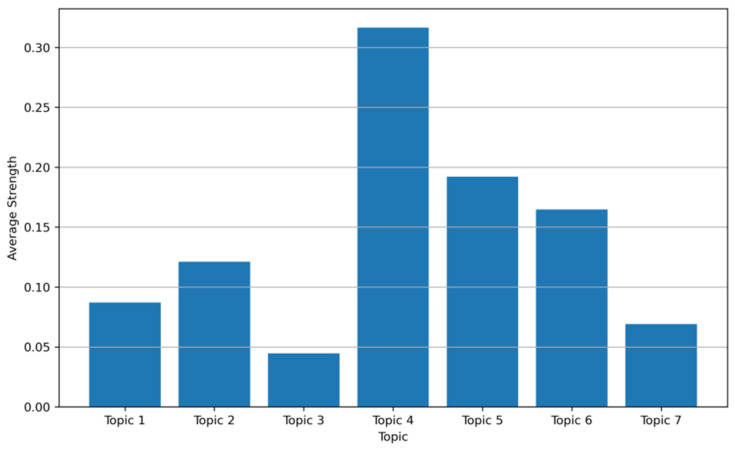
Average topic strength across the corpus.

As shown in Figure 9, the mean posterior probability of each topic across the corpus was calculated to represent its average topic strength, thereby providing a quantitative estimate of relative thematic prominence. Topic 4 (balance and gait training interventions, approximately 0.32) showed the highest strength, indicating that it is the most prominent topic in the corpus. This was followed by Topic 5 (evidence synthesis and review-based evaluation, approximately 0.19) and Topic 6 (elderly exercise and cognitive interventions, approximately 0.17), both of which also showed substantial thematic presence. Topic 2 (post-stroke upper-limb motor rehabilitation, approximately 0.12) and Topic 1 (vestibular rehabilitation and immersive training, approximately 0.09) occupied intermediate positions, reflecting continued attention to established rehabilitation-oriented applications. By contrast, Topic 7 (skill-oriented virtual task training, approximately 0.07) and Topic 3 (efficacy and adverse-effect assessment, approximately 0.05) showed lower average strengths, suggesting that these themes are less represented in the current corpus, although they may still reflect more specialized or developing lines of research. Overall, these results complement the qualitative interpretation of the LDA topics by clarifying which themes currently occupy the greatest share of the literature and which remain comparatively smaller within the broader thematic structure.

#### 3.2.4. Topic Temporal Evolution Analysis

**Figure 10:**
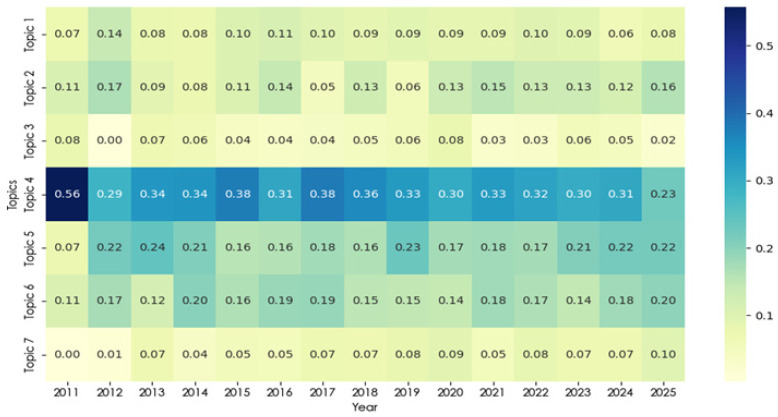
Topic strength heatmap over time (2011–2025).

**Figure 11:**
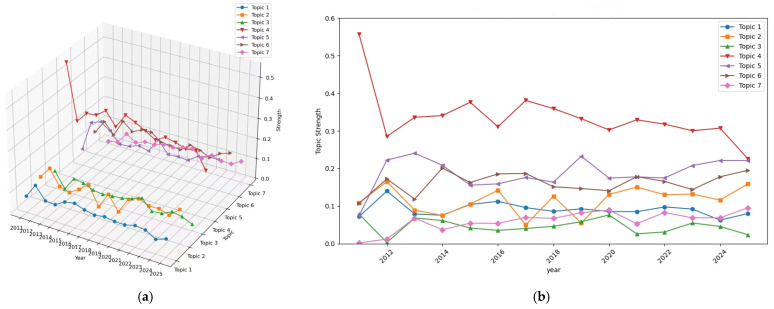
Topic strength over time. (**a**) Three-dimensional trend surface; (**b**) two-dimensional line chart.

Between 2011 and 2025, the topic strength heatmap and trend plots (Figure 10 and Figure 11) reveal clear differences in the temporal trajectories of the seven themes. Topic 4 (balance and gait training interventions) remained the most prominent theme throughout the study period. Although its strength was especially high at the beginning of the time series (approximately 0.56 in 2011), it subsequently stabilized at consistently elevated levels (generally around 0.30 or above), indicating that it has remained the dominant and most established area of research within the corpus.

By comparison, Topic 5 (evidence synthesis and review-based evaluation) and Topic 6 (elderly exercise and cognitive interventions) showed stronger representation from the early–mid 2010s onward and maintained relatively high levels in later years, reaching approximately 0.22 and 0.20, respectively, by 2025. This pattern suggests a sustained increase in the visibility of review-based evidence integration and aging-related intervention research within the broader field.

In contrast, Topic 3 (efficacy and adverse-effect assessment) showed a generally lower and declining profile over time, decreasing from earlier moderate values to approximately 0.02 by 2025. This suggests that, although evaluative and safety-related concerns remain present, they account for a comparatively smaller share of the literature in the later period. Topic 7 (skill-oriented virtual task training) started at a very low level but gradually increased after 2020, reaching approximately 0.10 by 2025, indicating growing visibility for task- and performance-oriented applications beyond traditional rehabilitation settings. This increase may also reflect broader technological changes in the field. In particular, the greater accessibility of immersive VR devices after 2020 may have made task-based and performance-oriented applications more feasible outside conventional rehabilitation contexts.

Topic 2 (post-stroke upper-limb motor rehabilitation) showed moderate but fluctuating strength across the study period, suggesting continued but variable attention, whereas Topic 1 (vestibular rehabilitation and immersive training) remained comparatively modest in strength across most years. Overall, the temporal patterns indicate that the field continues to be anchored by balance- and gait-related rehabilitation, while evidence synthesis, aging-related interventions, and skill-oriented training have become increasingly visible components of the thematic landscape in more recent years.

## 4. Discussion

### 4.1. From Feasibility to Differentiated Applications

The combined bibliometric and topic modeling results indicate that VR research in balance, coordination, and motor rehabilitation has moved from broad feasibility-oriented exploration toward a more differentiated and application-specific stage. In the earlier period, the literature was dominated by general terms related to virtual reality, exercise, balance, and rehabilitation, suggesting that the main concern was whether VR could function as a viable intervention tool. Over time, this broad orientation gave way to more targeted work, including vestibular rehabilitation, post-stroke upper-limb rehabilitation, aging-related interventions, and evidence synthesis [40,41,42]. In recent years, the field has shifted from general validation toward more specific adaptation to particular populations, intervention goals, and use contexts.

This shift also changes the central question in the field. The issue is no longer whether VR can be used in general, but which forms of VR intervention are most suitable for particular users, training aims, and implementation settings [43,44,45,46]. In other words, the field is moving from proof of feasibility toward proof of comparability, contextual fit, and practical relevance. The continued dominance of balance- and gait-related research confirms that the field still has a strong rehabilitation core, but the growing thematic diversification shows that this core is now accompanied by increasingly specialized application branches.

The collaboration and co-citation findings reinforce this interpretation. Country-level collaboration is relatively well connected, whereas institutional collaboration is more fragmented and concentrated around a smaller number of influential hubs. At the same time, the co-citation structure suggests that the field is supported by a coherent but diverse knowledge base spanning rehabilitation, game-based intervention, physical function training, and cognitive-related applications. Taken together, these patterns point to a field that is becoming more mature structurally while also expanding in thematic scope.

### 4.2. Dominant, Growing, and Emerging Thematic Lines

A key contribution of the present study is that it reveals a layered thematic structure rather than treating all identified topics as equally important. This distinction helps clarify not only what the field contains, but also how attention is distributed across established, expanding, and outward-moving areas of research.

The dominant thematic line is clearly balance and gait training interventions. This topic showed the highest average strength and remained the most prominent throughout the study period, indicating that balance-related rehabilitation continues to function as the central application area in the literature. This prominence likely reflects both clinical and technological factors. From a clinical perspective, impairments in balance, gait, and postural control are among the most common and functionally important rehabilitation targets across neurological, geriatric, and motor impairment populations. From a technological perspective, VR is particularly well suited to repetitive practice, feedback-guided postural training, and controlled motor task environments, which makes it especially compatible with balance- and gait-oriented intervention design. Together, these factors also suggest the relative maturity of this area, where balance, gait, and postural control have become established and repeatedly assessed outcomes [47,48,49].

A second layer includes growing themes such as evidence synthesis and review-based evaluation, as well as elderly exercise and cognitive interventions. The prominence of the evidence-synthesis theme suggests that the field has matured enough to support broader comparative reviews and review-based evaluation [50,51,52,53]. This theme is not simply a methodological by-product. It also reflects the growing need to organize and assess an expanding intervention literature. The rise in elderly exercise and cognitive interventions likewise suggests that VR research is moving beyond post-impairment recovery alone. It increasingly includes aging-related prevention, fall risk reduction, and combined physical–cognitive support. This shift broadens the functional scope of the field and points to more preventive and maintenance-oriented applications.

A third layer includes more specialized and outward-expanding themes, most notably skill-oriented virtual task training. Although this topic occupies a smaller share of the corpus than the dominant rehabilitation-focused themes, its increasing visibility suggests that the field is extending beyond traditional clinical rehabilitation into broader training-oriented contexts. This line of work appears particularly relevant to younger and other non-clinical populations. In these contexts, VR may support structured task practice, motor learning, and performance-related training rather than recovery alone [54,55]. Its importance lies not in current dominance, but in the way it marks a visible expansion of the field into new educational, exercise, and skill-development settings.

In contrast, the declining visibility of efficacy and adverse effect assessment suggests that this line of research occupies a smaller share of the literature in the later period. One possible explanation is that safety and evaluative issues are increasingly addressed within application-specific intervention studies rather than maintained as a distinct thematic line. However, another possibility should also be acknowledged: adverse effect monitoring may still be underrepresented in VR rehabilitation research. This is clinically important because lower thematic visibility does not necessarily mean that safety concerns are being consistently monitored or adequately reported.

Overall, these results suggest that the field is no longer organized around a single undifferentiated agenda. Instead, it consists of a stable rehabilitation core, several expanding branches, and a smaller but meaningful set of outward-moving themes that connect VR-based motor work to broader training and non-clinical applications.

### 4.3. Added Value of Integrating Bibliometric Mapping and Topic Modeling

A further contribution of this study lies in its methodological integration. Conventional descriptive summaries and narrative reviews can synthesize findings within specific populations or intervention types, but they are less suited to capturing the broader structural evolution of a field that spans multiple application settings and levels of evidence. By combining bibliometric mapping with topic modeling, the present study offers a more structured explanation of how the literature has developed at both the macro and micro levels.

At the macro level, CiteSpace and VOSviewer make it possible to identify publication trends, collaboration patterns, keyword relationships, co-citation structures, and broad temporal shifts. These analyses clarify how the field is organized socially and intellectually. At the micro level, LDA topic modeling complements this by identifying recurring semantic themes, estimating their relative prominence, and tracing their temporal trajectories. This moves the analysis beyond visible keyword frequency alone and toward a more interpretable view of latent thematic organization.

The value of combining these approaches lies not simply in using multiple tools, but in connecting field-level evolution with topic-level differentiation. In the present study, the broad transition identified through keyword evolution—from early validation to more targeted applications—was consistent with the LDA-based pattern of dominant, growing, and emerging themes. More concretely, broad bibliometric patterns centered on rehabilitation, balance, gait, exercise, and older adults were reflected at the LDA level in more specific themes such as balance and gait training interventions and elderly exercise and cognitive interventions. Similarly, the broader rehabilitation-oriented core identified through keyword and co-citation structure was further differentiated by LDA into post-stroke upper-limb rehabilitation and vestibular rehabilitation and immersive training. These correspondences reinforce the view that bibliometric analyses identify the macro-level structure of the field, whereas LDA helps clarify how that structure is internally organized into more focused and interpretable research directions. This convergence strengthens the interpretation of the findings because the macro-level and topic-level analyses support the same developmental narrative.

Accordingly, the main added value of this study is not that it identifies more topics than a conventional review, but that it clarifies how a fragmented body of literature is becoming internally structured. By showing where research is most concentrated, where it is expanding, and where it is beginning to move outward into new contexts, the present study provides a more structured overview than conventional descriptive summaries and offers a clearer account of the field’s developmental logic.

### 4.4. Implications for Study Design and Evidence Synthesis

The findings of this study have several implications for future study design and for how evidence in this field should be synthesized. The most immediate implication is that increasing thematic diversity now makes cross-study comparability a central challenge. As VR applications become more differentiated across rehabilitation, aging-related intervention, and non-clinical training contexts, variation in participants, intervention aims, and outcome priorities makes it harder to compare findings unless reporting becomes more consistent.

A first priority is therefore clearer and more standardized reporting of intervention characteristics. Across the dominant and growing themes, several features recur repeatedly, including task-oriented practice, graded difficulty, feedback-based training, and different levels of immersion. Future studies would benefit from reporting these components more explicitly, including feedback type, progression logic, session structure, training dose, and contextual setting. Greater consistency in these areas would improve reproducibility and make comparisons across studies more meaningful [52,56,57,58].

A second implication is the need to distinguish more clearly between rehabilitation-focused and broader training-oriented applications. As the field extends beyond clinical rehabilitation into more diverse forms of motor practice, especially in younger or other non-clinical populations, intervention goals are becoming increasingly heterogeneous. Some studies aim to restore impaired function, whereas others focus on skill acquisition, movement quality, participation, or preventive exercise. These aims are not interchangeable, and clearer conceptual separation between them would improve outcome selection, reduce overgeneralization, and support more coherent synthesis [59,60,61,62].

A third implication is that evidence synthesis will become increasingly important as the literature continues to expand. The prominence of the evidence-synthesis theme indicates that the field is now large enough to support broader comparative reviews, but it also suggests that future syntheses must be more sensitive to thematic heterogeneity. Reviews should pay closer attention to differences in population type, intervention purpose, and study design rather than treating VR-based interventions as a single homogeneous category. In this respect, the present study may help identify which parts of the field are sufficiently mature for deeper synthesis and which remain too heterogeneous for reliable aggregation [18,63,64,65].

These distinctions further suggest that future VR interventions should be designed with closer attention to target population, intervention purpose, and intended functional outcome, since the field now spans not only rehabilitation-focused applications but also broader training- and skill-oriented contexts.

### 4.5. Limitations and Directions for Future Research

Several limitations should be considered when interpreting the findings of this study. First, the analysis was based on records indexed in the Web of Science Core Collection and limited to English-language publications. Although this provided a consistent and analyzable corpus, relevant studies indexed in other databases or published in other languages may have been underrepresented. The present evidence map should therefore be understood as a structured view of a substantial segment of the literature rather than a fully exhaustive account of all global work in this area [66,67].

Second, the findings are necessarily shaped by the search strategy used to define the dataset. In particular, the inclusion of terms such as “sport” and “exercise” helped capture training-oriented and non-clinical applications of VR, but it may also have favored literature framed in exercise or sport-related language over other relevant rehabilitation studies using different terminology. As a result, the dataset may not fully represent all segments of the broader VR rehabilitation literature, and the findings should be interpreted within this search boundary. In addition, as with other topic modeling studies, the thematic structure is influenced to some extent by preprocessing and model specification, although the seven topic solution was selected based on coherence, perplexity, and consistency with the broader bibliometric patterns [57,68,69]. A formal sensitivity analysis with and without the “sport/exercise” constraint was not conducted in the present study. In addition, beyond the coherence- and perplexity-based evaluation used for topic-number selection, no further formal robustness checks were performed for the LDA solution. The resulting thematic structure should therefore be interpreted cautiously.

Third, the temporal analysis was conducted at an annual level, which is appropriate for broad developmental interpretation but may obscure shorter-term fluctuations or transitional changes within specific subtopics. This is especially relevant in a field influenced by rapid changes in devices, software, and application settings, where thematic movement may not always follow smooth year-to-year trajectories [70,71].

These limitations also suggest several focused directions for future research. Future studies could broaden coverage by incorporating additional databases and multilingual sources. It would also be valuable to test the robustness of the thematic structure under alternative preprocessing pipelines and topic specifications, particularly for smaller or more specialized themes. Finally, more refined temporal modeling may help capture subtler shifts in how particular intervention domains emerge, stabilize, or branch into new applications over time. Such extensions would strengthen future evidence maps and provide an even clearer account of how VR research in balance, coordination, and motor rehabilitation continues to evolve [72,73,74].

## 5. Conclusions

This study combined bibliometric mapping and LDA topic modeling to examine the evolution of VR research in balance, coordination, and motor rehabilitation from 2011 to 2025. The findings show that the field has moved beyond early feasibility-focused work toward a more differentiated structure shaped by distinct population groups, intervention purposes, and application contexts. Balance- and gait-related interventions remained the most established line of research, while evidence synthesis, aging-related interventions, and skill-oriented training applications gained increasing visibility.

Taken together, these results suggest that the field is no longer defined mainly by whether VR can be used, but by how it is being tailored to specific rehabilitation and training needs. Future studies should therefore place greater emphasis on clearer intervention reporting, more context-sensitive study design, and more targeted evidence synthesis across different application domains. Overall, the present study provides a descriptive mapping of the field and a more structured overview of how VR research on balance, coordination, and motor rehabilitation has developed across populations, intervention types, and application contexts.

## Figures and Tables

**Table 1:** Top 15 keywords in the co-occurrence network.

Keyword	Frequency	Betweenness Centrality	First Appearance Year
virtual reality	549	0.04	2011
exercise	377	0.11	2011
balance	327	0.12	2011
rehabilitation	226	0.08	2011
older adults	193	0.13	2011
physical activity	152	0.10	2011
gait	151	0.04	2013
people	127	0.06	2012
performance	122	0.05	2012
reliability	121	0.10	2011
quality of life	120	0.03	2015
parkinsons disease	107	0.06	2012
program	88	0.06	2012
therapy	84	0.08	2012
falls	80	0.06	2012

## Data Availability

The search strategy and analytical procedures are described in the article. Data used in this study were retrieved from the Web of Science Core Collection. Processed data supporting the findings are available from the author upon reasonable request.

## Supplementary Materials

The following supporting information can be downloaded at: https://www.mdpi.com/article/10.3390/healthcare14081067/s1, Table S1. Top 10 terms with weights for each LDA topic.

## Abbreviations

The following abbreviations are used in this manuscript:

VR	Virtual reality
LDA	Latent Dirichlet Allocation
WoSCC	Web of Science Core Collection
WoS	Web of Science
LLR	Log-likelihood ratio
C_v	Coherence score
